# Clinical–radiomics model for predicting internal mammary lymph node metastasis in operable breast cancer patients

**DOI:** 10.3389/fonc.2025.1477866

**Published:** 2025-04-03

**Authors:** Wei Wang, Wenyu Zhang, Ting Yu, QingWei Wu, ChengLin Yang, Jianbin Li

**Affiliations:** ^1^ Department of Radiation Oncology, Shandong Cancer Hospital and Institute, Shandong First Medical University and Shandong Academy of Medical Sciences, Jinan, Shandong, China; ^2^ Department of Radiology, Shandong Cancer Hospital and Institute, Shandong First Medical University and Shandong Academy of Medical Sciences, Jinan, Shandong, China; ^3^ Cancer Center, Shandong Provincial Hospital Affiliated to Shandong First Medical University, Jinan, Shandong, China; ^4^ Department of Nuclear Medicine, Shandong Cancer Hospital and Institute, Shandong First Medical University and Shandong Academy of Medical Sciences, Jinan, Shandong, China; ^5^ Department of Radiation Oncology, Yantai Yuhuangding Hospital affiliated to Qingdao University, Yantai, Shandong, China

**Keywords:** breast cancer, internal mammary node metastasis, clinicopathological factors, DCE-MRI radiomics, predictive model

## Abstract

**Objective:**

Although preoperative prediction of axillary lymph nodes status has been achieved using radiomics and combined models, there is a dearth of research on internal mammary lymph node (IMN) metastasis status prediction. We developed a predictive model by combining clinicopathological factors with preoperative dynamic contrast-enhanced magnetic resonance imaging (DCE-MRI) radiomics to accurately predict IMN metastasis in breast cancer.

**Methods:**

Patients who had no evidence of IMN metastasis on preoperative images but underwent internal mammary sentinel lymph node biopsy (IM-SLNB) were included in this study. Preoperative DCE-MRI and clinicopathological data of 124 patients with breast cancer were obtained, to developed Clinical, radiomics, and clinical–radiomics models, separately. Decision curve analysis (DCA) was employed to assess the models’ clinical applicability.

**Results:**

The resulting area under the curves (AUCs) were 0.913, 0.831, 0.964 for the clinical model, the radiomics model, and the clinical–radiomics model, respectively. The Delong test revealed significant differences in the receiver operating characteristic (ROC) curves only between the clinical and clinical–radiomics models (all P<0.05). DCA substantiated the clinical–radiomics model’s optimal predictive efficiency, enhanced discriminatory ability, and maximum benefit. The AUC (95% confidence interval: 0.935-0.993) of the clinical–radiomics model is 0.964. Repeated k-fold cross validation showed that average accuracy and Standard deviation of clinical–radiomics model are 90.23% and 8.45%, respectively. And the calibration slope of clinical–radiomics model is 1.08(p=0.071).

**Conclusions:**

Although the clinical model was effective in predicting IMN status, the addition of DCE**-**MRI radiomics significantly improved the predictive value of the clinical–radiomics model, which showed excellent discrimination, calibration, and stability. This suggests that the clinic-radiomics model has potential for preoperative assessment of IMN metastasis risk in breast cancer patients, but external validation is needed to confirm its clinical utility. IMN irradiation is recommended for early patients with high IMN metastasis risk, and overtreatment should be avoided for patients with low metastasis risk.

## 1 Introduction

Precise staging of lymph node metastasis forms an integral part of breast cancer staging and serves as a prerequisite for accurate determination of the treatment strategy and prognostication (1). The internal mammary lymph nodes (IMN) are situated posterior to the intercostal muscles and costal cartilage, adjacent to the internal mammary veins and arteries and receive lymphatic drainage from the nipple-areola complex, medial aspect of the breast, anterior chest wall, precostal pleura, and upper abdominal wall (2, 3). IMNs constitute a significant pathway for the lymphatic spread of breast cancer. The IMN metastasis rate was 5-17% in axillary lymph node (ALN)-negative patients and 28-52% in ALN-positive patients (4–7). Studies have revealed that the prognostic value of IMN metastasis is comparable to that of ALN metastasis (8, 9). The presence of IMN metastasis significantly increases the likelihood of distant metastasis compared to the absence of IMN involvement (6, 10–12). Therefore, accurate assessment of IMN status is crucial for precise therapeutic decision-making and achieving favorable outcomes.

The most critical aspect of detecting metastasis in IMNs is identifying suspicious lymph nodes on imaging tests such as ultrasound, MRI, or PET. However, owing to the complex anatomy of the internal mammary region, the diagnostic accuracy rates of clinical examination and imaging are limited. Therefore, despite advances in precision medicine, pathological examination remains the gold standard for IMN diagnosis. Extended radical mastectomy (ERM) has been discarded as a treatment option since it is extremely traumatic and lacks survival benefits (13). Minimally invasive approaches for lymph node management, internal mammary sentinel lymph node biopsy (IM-SLNB), and video-assisted thoracic surgery have emerged as alternatives, owing to the widespread popularity of SLNB (6, 14). However, these procedures are invasive with potential complications, can only be performed once at a specific spatial location, and cannot be repeated. Therefore, it is necessary to explore effective noninvasive methods for assessing the status of the IMN and identifying patients at high risk of IMN metastasis who would benefit from postoperative radiotherapy, while avoiding unnecessary treatment/intervention in the IMN drainage area for low-risk patients.

Although predictive models for noninvasive ALN status assessment in clinical practice are being developed gradually, few studies have investigated IMN metastasis prediction (15–20). Hence, this study aimed to establish a clinical and radiomics model based on clinicopathological factors and dynamic contrast-enhanced magnetic resonance imaging (DCE-MRI) radiomics characteristics and combine them to construct a clinical–radiomics model, compare the predictive efficiency of different models for IMN metastasis, and provide a basis for individualized decision-making in IMN radiotherapy after breast cancer surgery.

## 2 Methods

### 2.1 Patients

This study enrolled patients with operable non-advanced breast cancer without IMN metastasis on preoperative imaging who underwent IM-SLNB with or without IMN dissection and treatment at Shandong Cancer Hospital from January 2013 to December 2019. The preoperative DCE-MRI scans and preoperative clinicopathological data were collated. The inclusion criteria were as follows: (i) patients newly diagnosed with operable non-stage IV breast cancer, (ii) absence of IMN metastasis confirmed on preoperative diagnostic imaging, (iii) patients who underwent IM-SLNB with or without IMN dissection, and (iv) complete preoperative DCE-MRI data for primary breast tumors. The exclusion criteria were patients who received neoadjuvant therapy, patients with regional lymph node metastasis except for ALN and IMN, and patients with incomplete clinicopathological or imaging data.

All patients included in this study had both preoperative DCE-MRI images and complete clinicopathological data. To ensure data consistency, we carefully matched each patient’s imaging data with their corresponding clinical records before feature extraction and model construction. This approach eliminated any potential inconsistencies in the number of instances between the image-derived dataset and the clinical dataset.

The requirement for written informed consent from patients was waived due to the retrospective nature of the investigation (retrospective single-institution cohort study). The institutional research ethics board of Shandong Cancer Hospital and Institute approved this study (SDTHEC201703014), and all methods were performed in accordance with the relevant guidelines and regulations.

### 2.2 Clinicopathological data collection

The patients’ clinicopathological data included age, primary tumor location, presence or absence of tumor thrombus, menstrual status, pathological type, histological grade, estrogen receptor (ER) status, progesterone receptor (PR) status, human epidermal growth receptor (HER)-2 expression level, Ki67 expression level, molecular subtype, number of ALN metastasis (ALN), and T stage. And all of the pathological data obtained from samples obtained after surgery.

### 2.3 DCE-MRI acquisition

The preoperative DCE-MRI images of all patients were collected. All MR images were acquired using a Philips Achieva 3.0-T MR scanner with the patient in the prone position, with both breasts falling naturally. The scanning range encompassed both breasts and axillary soft tissues. The complete MRI sequence included bilateral breast cross-sectional T1-weighted imaging, sagittal T2-weighted imaging with fat suppression, diffusion-weighted imaging, and DCE scanning using gadopentetate meglumine as the contrast medium for enhanced imaging. The following DCE-MRI parameters were used: repetition time=4.4 ms; echo time= 2.2 ms; field-of-view=34 cm × 34 cm; slice thickness=4.0 mm; and flip angle=12°. In this study, DCE was divided into eight phases, with tumor enhancement being the most prominent in the second phase. Therefore, segmentation and analysis were performed on the second-phase DCE images. Subsequently, continuous preoperative DCE-MRI images were imported into a deformation registration software called 3D Slicer (version 4.13.0, http://www.slicer.org, USA).

### 2.4 Establishment of the clinical prediction model

Continuous variables such as age, preoperative neutrophil count, preoperative lymphocyte count, and preoperative monocyte count were entered into the univariate analysis of clinicopathological factors. Student’s t-test or Mann-Whitney U test were used for the analysis. Categorical variable included primary tumor location, presence or absence of tumor thrombus, menstrual status, pathological type, histological grade, ER status, PR status, HER-2 expression, Ki-67 expression, molecular classification, number of ALN metastasis, and T stage. Analysis was performed using the chi-squared or Fisher’s exact test. A clinical predictive model was established based on the independent risk factors for IMN metastasis identified by univariate and multivariate logistic regression analysis. Variables with p-values < 0.1 in the univariate analysis were included in the multivariate logistic regression analysis. Among these, only variables with p-values < 0.05 in the multivariate analysis were retained to establish the final prediction model.

### 2.5 Establishment of the radiomics model

**Figure 1:**
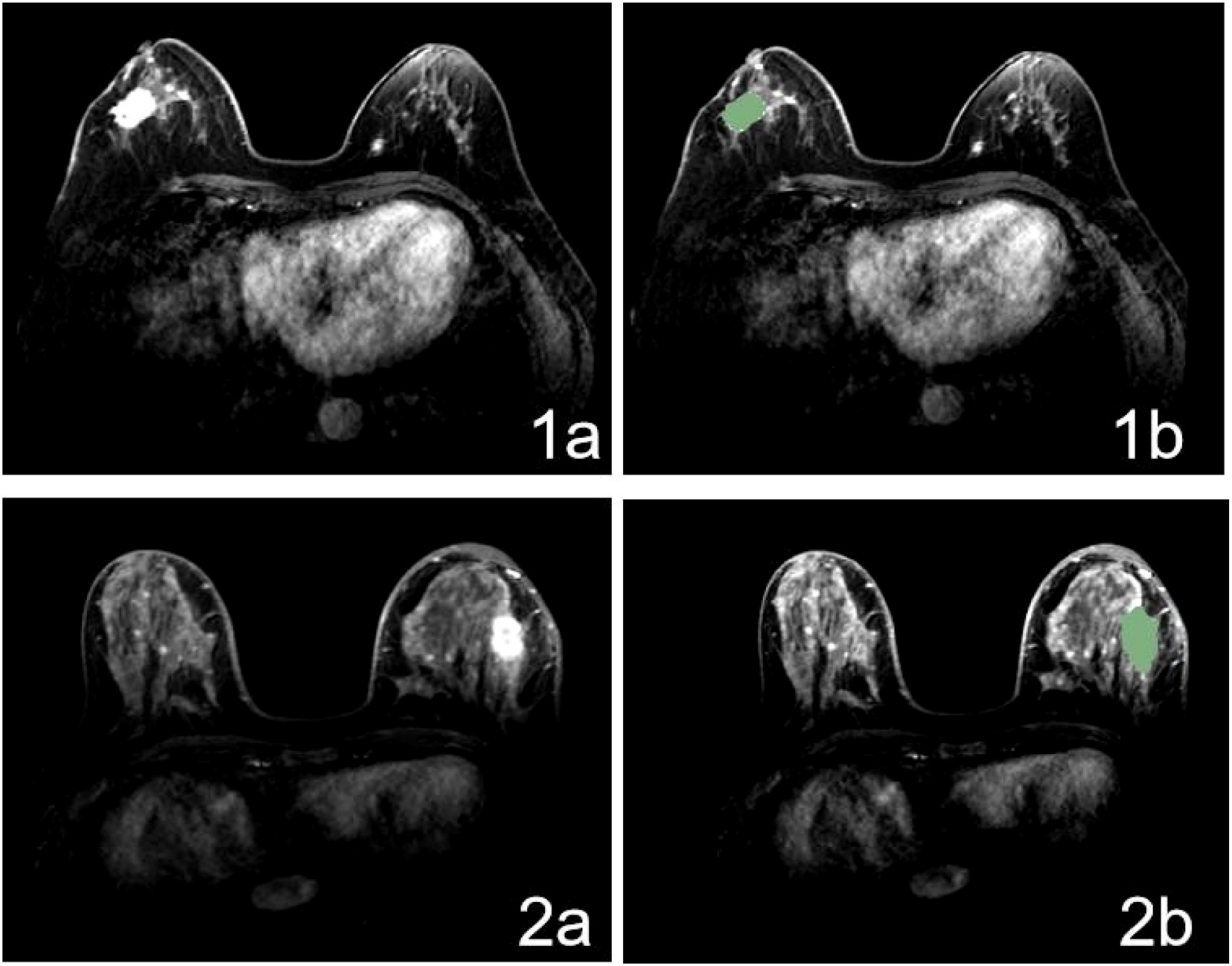
MR Image for patient with positive **(1a)** and negative **(2a)** IMLN and ROI delineating based on DCE-MR image **(1b, 2b)**.

The process of radiomics analysis consists of segmentation of the regions-of-interest (ROIs) on MRI (**Figure 1**); image pre-processing, feature extraction, and selection of radiomics; and establishment of a radiomics model.

#### 2.5.1 ROI segmentation on DCE-MRI

Using 3D Slicer, the ROI was segmented on the second-enhancement phase of preoperative diagnostic MRI. The ROI was manually delineated by a radiation oncologist blinded to the patients’ IMN status. All ROIs were examined and evaluated by another radiation oncologist with over 10 years’ experience. In this study, the primary breast tumor served as the ROI, whose contour was meticulously delineated layer-by-layer along its boundary, excluding the adjacent blood vessels, fat tissue, and normal structures, while minimizing the inclusion of edematous areas surrounding the tumor.

#### 2.5.2 Image pre-processing, feature extraction, and radiomics feature selection

Before feature extraction, each image was subjected to pre-processing. The Caret package in R software was utilized for data pre-processing, which facilitates faster algorithm convergence and yields a more reasonable model. To reduce noise and enhance image quality, we applied Gaussian smoothing to the DCE-MRI images. This technique helps to suppress high-frequency noise while preserving the structural details of the tumor. Intensity normalization was performed to standardize the voxel values across different images. This step ensures that the radiomics features are comparable across patients, reducing variability caused by differences in imaging protocols or scanner settings. 3D Slicer was used for voxel-based radiomics feature extraction, encompassing first-order statistics, and shape and texture features. Texture features effectively capture tumor heterogeneity by utilizing a gray-level dependence matrix, gray-level run-length matrix, gray-level co-occurrence matrix, gray-level size zone matrix, and neighborhood gray tone difference matrix. Feature selection plays a pivotal role in training classifiers because it reduces the computational complexity while enhancing the classification accuracy. This process entails evaluating the linear correlation between each feature and category label, followed by eliminating variables with strong correlations with other independent variables and those exhibiting multicollinearity. Subsequently, data centralization was performed along with standard deviation normalization [(x-mean)/SD)]. Finally, the most relevant features were selected from the entire set using the least absolute shrinkage and selection operator (LASSO) logistic regression method, followed by the forward method based on partial maximum likelihood estimation, to identify the best predictive features related to IMN metastasis prediction.

#### 2.5.3 Establishment of the radiomics score

After feature selection, a radiomics signature, also known as the Radiomics Score (RS), was created from a linear combination of features and corresponding weights, and calculated as RS=β0 + β1×1 + β2X2 +… + βnXn + compensation coefficient, where β0 is a constant, βi is the logistic regression coefficient, and Xi is the value of the selected feature. The RS, which reflects the risk of IMN metastasis, was calculated for each patient using a linear combination of selected features weighted by the respective coefficients.

### 2.6 Establishment of the clinical-radiomics model

To integrate the radiomics features with the clinicopathological factors, we first calculated the RS based on the selected radiomics features of their respective coefficients. The RS was then combined with the independent clinicopathological risk factors identified through multivariate logistic regression. The combined model was used to construct a nomogram for predicting the risk of IMN metastasis. The integration of these two datasets allowed us to leverage both imaging and clinical information to improve the predictive accuracy of the model.

### 2.7 Model testing and comparison

The Delong test was employed to compare the performance of the receiver operating characteristic (ROC) curves across different models. It evaluates whether the differences in predictive performance between models are statistically significant by calculating the variance and covariance of the AUC values. Decision curve analysis (DCA) was used to assess the clinical applicability of the three models, ultimately determining the optimal prediction model for IMN metastasis risk. Model goodness-of-fit was evaluated using the Hosmer–Lemeshow test and calibration curve. The validity, calibration and stability of the model were verified using repeated k-fold CV(cross-verification). The number of cross-verification folds is 5(k=5). And then performed 100 replicate cross-validation times, each time using a different random seed for data division.

### 2.8 Statistical analysis

Statistical analyses were conducted using SPSS 26.0, and R (version 3.6.0, http://www.r-project.org) software packages. The LASSO logistic regression method was employed to identify the most discriminative features. Radiomics features were computed by combining their weighted coefficients. Cross verification of the clinical–radiomics model was performed using repeated k-fold CV. Model performance evaluation included ROC analysis, calibration curve assessment, and DCA. Differences in the ROC curves among the three models were compared using the Delong test, which is a significance test for area under the curve (AUC) values obtained by calculating the variance and covariance across different ROC curves. Generally, p-values<0.05 indicated statistically significant differences between AUC values from two ROC curves tested at an α level of 0.05.

## 3 Results

### 3.1 Patients’ characteristics

124 patients were included in the final analysis. The baseline characteristics of all patients are presented in **Table 1**. Twenty-one patients had IMN metastasis and 103 did not. The overall incidence of primary tumors in the medial quadrant was 38.7%, 20.8% of which exhibited IMN metastases. Eight patients with IMN metastasis had lateral quadrant primary tumors and three had central region primary tumors. IMN metastasis occurred in the medial quadrant, lateral quadrant, and central region in 20.8%, 11.8%, and 37.5% of patients, respectively. IMN metastasis occurred in 90.5% and 9.5% patients in the ALN-positive and ALN-negative groups, respectively.

**Table 1:** Clinicopathological characteristics of the patients.

Clinicopathologic characteristics	IMN metastasis No. (%)	No IMN metastasis No. (%)	All No. (%)
IMN	21 (16.9)	103 (83.1)	124
Age			
≤50	13 (18.9)	56 (81.1)	69 (55.6)
>50	8 (14.5)	47 (85.5)	55 (44.4)
Primary tumor location			
medial	10 (20.8)	38 (79.2)	48 (38.7)
lateral	8 (11.8)	60 (88.2)	67 (54.8)
central	3 (37.5)	5 (62.5)	8 (6.5)
Vascular invasion			
yes	5 (33.3)	10 (66.7)	15 (12.1)
no	16 (14.7)	93 (85.3)	109 (87.9)
Menstrual status			
postmenopausal	7 (13.5)	45 (86.5)	52 (41.9)
premenopausal	14 (19.4)	58 (80.6)	72 (58.1)
Pathological subtypes			
ductal	21 (17.8)	97 (82.2)	118 (95.2)
lobular	0	1 (100)	1 (0.8)
other	0	5 (100)	5 (4.0)
Histological grade			
I	0	2 (100)	2 (1.6)
II	14 (19.7)	57 (80.3)	71 (57.3)
III	7 (14.0)	43 (86.0)	50 (40.3)
uncertain	0 (0)	1 (100)	1 (0.8)
ER			
positive	19 (20.7)	73 (79.3)	92 (74.2)
negative	2 (6.3)	30 (93.7)	32 (25.8)
PR			
positive	19 (25.0)	57 (75.0)	76 (61.3)
negative	2 (4.2)	46 (95.8)	48 (37.5)
HER-2			
positive	16 (18.4)	71 (81.6)	87 (70.2)
negative	5 (13.5)	32 (86.5)	37 (29.8)
Ki-67			
high expression	12 (13.8)	75 (86.2)	87 (70.2)
low expression	9 (24.3)	28 (75.7)	37 (29.8)
Molecular subtypes			
luminal A	12 (24.0)	38 (76.0)	50 (40.3)
luminal B	3 (17.6)	14 (82.4)	17 (13.7)
her2-enriched	5 (13.2)	33 (86.8)	38 (30.7)
three negative	1 (5.3)	18 (94.7)	19 (15.3)
Number of ALN metastases			
0 (N0)	2 (2.9)	68 (97.1)	70 (56.5)
1-3 (N1)	9 (24.3)	28 (75.7)	37 (29.8)
4-9 (N2)	4 (44.4)	5 (55.6)	9 (7.2)
>10 (N3)	6 (75.0)	2 (25.0)	8 (6.5)
T grade			
T1	8 (13.1)	53 (86.9)	61 (49.2)
T2	11 (20.8)	42 (79.2)	53 (42.7)
T3	2 (22.2)	7 (77.8)	9 (7.3)
T4	0	1 (100)	1 (0.8)

IMN, internal mammary lymph node; ER, estrogen receptor; PR, progesterone receptor; HER-2, human epidermal growth factor receptor 2.

### 3.2 Clinical model

**Figure 2:**
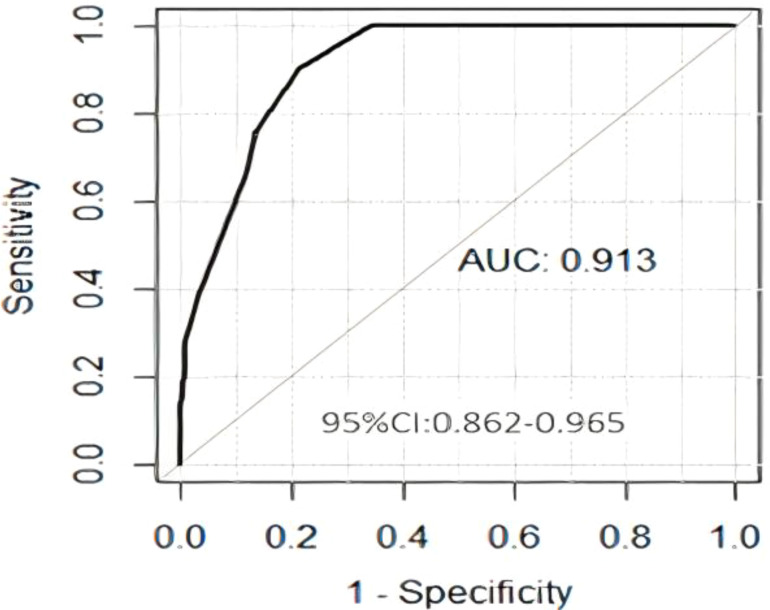
ROC curve of the clinical model. ROC, receiver operating characteristic.

Univariate logistic regression analyses and multivariate logistic regression analyses (**Table 2**) revealed that primary tumors located in the medial quadrant, positive PR status, and ALN metastasis were independent risk factors for IMN metastasis. Subsequently, a clinical prediction model was developed based on tumor location, PR status, and ALN metastasis status. ROC curve analysis showed that the AUC for predicting IMN metastasis was 0.913 (95% confidence interval [CI]: 0.862-0.965) (**Figure 2**).

**Table 2:** Univariate and multivariate logistic analysis.

Clinicopathologic characteristics	Univariate logistic analysis			Multivariate logistic analysis		
	p	OR	95%CI	p	OR	95%CI
Age	0.668	0.99	0.94~1			
Tumor location						
lateral quadrant						
medial quadrant	0.096	0.44	0.17~1	0.019	0.17	0.04~0.75
Vascular invasion	0.081	2.91	0.88~9	0.233	0.30	0.04~2.16
Menstrual status	0.383	0.64	0.24~1			
Pathological subtypes						
ductal						
lobular	0.997	0.00	0.00~Infinite			
other	0.993	0.00	0.00~Infinite			
Histological grade						
I	1.000	1.00	0.00~Infinite			
II	0.995	3912840	0.00~Infinite			
III	0.995	2547896	0.00~Infinite			
ER	0.079	3.90	0.86~1	0.805	0.71	0.05~10.64
PR	0.008	7.67	1.70~3	0.035	12.14	1.20~122.88
HER-2	0.509	0.69	0.23~2			
Ki-67	0.157	0.50	0.19~1			
Molecular subtypes						
luminal A						
luminal B	0.589	0.68	0.17~2 2.77			
her2-enriched	0.208	0.48	0.15~1 1.50			
three negative	0.107	0.18	0.02~1 1.46			
Number of ALN metastases	<0.001	4.37	2.37~8	0.000	8.63	3.25~22.9222.9
T grade						
T1						
T2	0.992	116998231701111	0.00~Infinite			
T3	0.992	17017924	0.00~Infinite			
T4	1.00	1.00	0.00~Infinite			

### 3.3 Radiomics model

**Figure 3:**
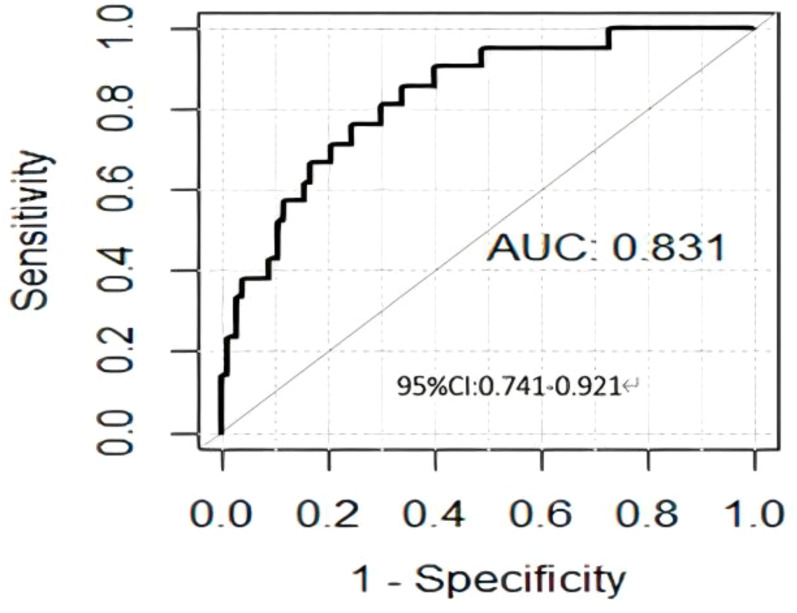
ROC curve of the radiomics model. ROC, receiver operating characteristic.

A total of 850 radiomic features were extracted from each patient. Features with a correlation coefficient greater than 0.9 were eliminated, and 185 features remained. Further elimination of collinear features reduced the number to 123 and subsequent rank-sum testing further narrowed the number to 122. Following LASSO feature selection, four radiomics features with nonzero coefficients were ultimately chosen to construct the radiomics model: one shape feature (original_shape_Elongation) and three texture features (wavelet-LHL_glcm_Correlation, wavelet-LHL_glcm_Correlation, and wavelet-LHH_ngtdm_Contrast). These features were utilized to establish a predictive radiomics model, where the RS was calculated using the following formula: RS=0.730 × original_shape_Elongation + 0.916 × wavelet-LHL_glcm_Correlation + 1.271 × wavelet-LHL_ first order _Skewness - 1.267 × wavelet-LHH_ngtdm_Contrast - 2.305. Performance evaluation yielded an AUC value of 0.831 (95%CI: 0.741-0.921) (**Figure 3**).

### 3.4 Clinical-radiomics model

**Figure 4:**
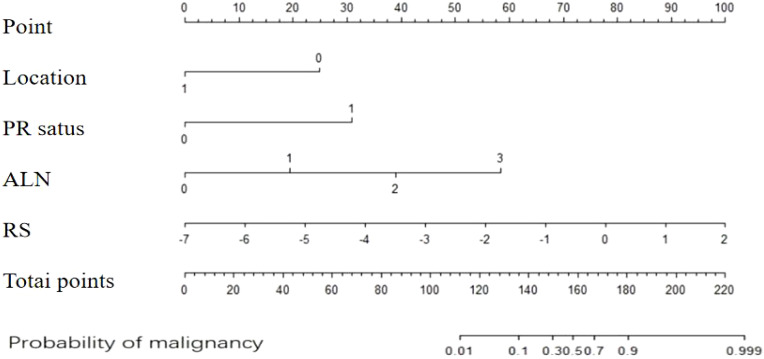
Nomogram for predicting the risk of IMN metastasis based on clinical-radiomics. IMN, internal mammary lymph node.

**Figure 5:**
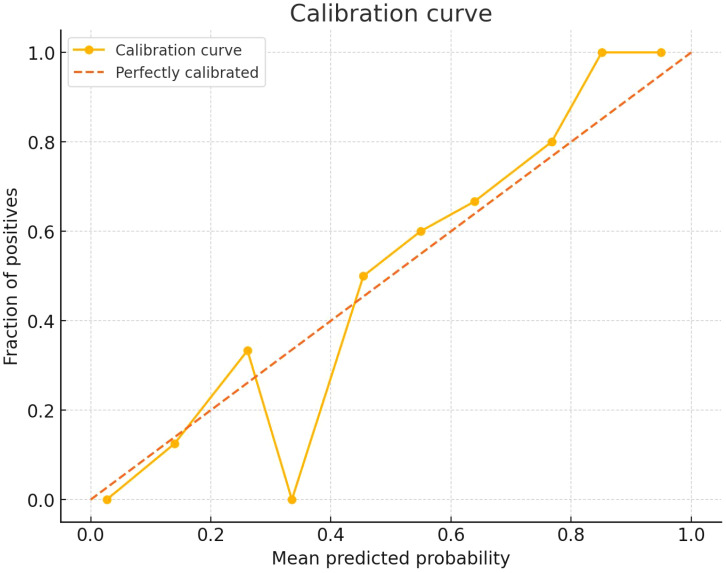
Calibration curve of the clinical–radiomics model.

The clinical–radiomics prediction model was established using the selected clinicopathological features and RS. To enhance its clinical applicability and provide a more intuitive representation, we established a nomogram (**Figure 4**), whose AUC for predicting IMN metastasis was 0.964 (95%CI: 0.935-0.993). As shown in **Figure 5**, the calibration curves of the model was plotted. The x-axis represents the predicted risk, and the y-axis represents the actual probability. The diagonal dashed line represents a perfect prediction by an ideal model and the orange curve represents the performance of the model, of which a closer fit to the diagonal dashed line represents a better prediction. But the orange curve dips below the reference line, which means the predicted probability is higher than the actual probability, that is, the model has overestimated the actual probability between 0.2 and 0.4. We calculate the calibration curve of the model, and use the slope of the calibration curve to evaluate the match between the probabilistic predictions of the model and the actual results. It is found that the prediction model has bias or error in the range between 0.2 and 0.4. The calibration slope is 1.08 (p=0.071), indicating no statistically significant difference from 1. And it indicates that the predicted probability of this model matches well with the actual event. The mean, standard deviation, minimum and maximum of the calibration slopes from repeated cross-validation were 1.076, 0.023, 1.034 and 1.123, respectively. Ideally, the calibration slope should be close to 1, and a value of 1.08 indicates that the model’s prediction probability is slightly high, but still performs relatively accurate overall. The calibration curve demonstrated strong concordance between the probability predicted by the clinical–radiomics model and the actual rate of IMN metastasis in patients (**Figure 5**).

### 3.5 Comparison and testing of the three prediction models

**Figure 6:**
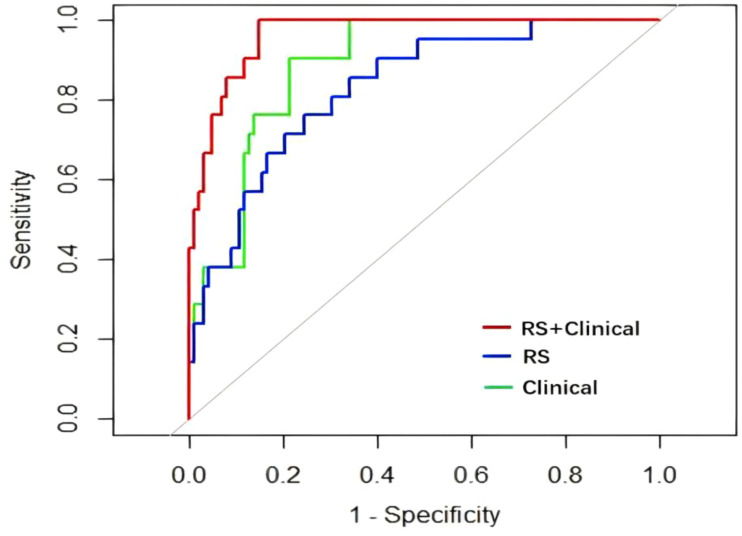
Comparison of ROC curves of the three models. RS, radiomics model; Clinical, clinical model; RS+Clinical, clinical–radiomics model.

**Figure 7:**
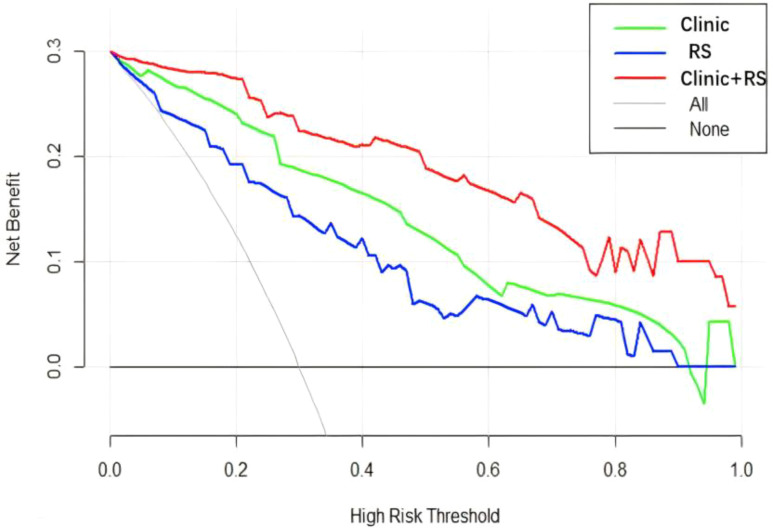
Decision curve analysis.

The Delong test did not identify significant differences in the ROC between the clinical and radiomics models (Z=1.4996, p=0.134), but found significant differences between the clinical and clinical–radiomics models (Z=-2.4294, p=0.015) and between the radiomics and clinical-radiomics models (Z=-3.252, p=0.001). Based on the AUC of the three models, the clinical–radiomics model provided the most efficient prediction for the IMN status (**Figure 6**). DCA was performed to visually demonstrate the differences between the models and their clinical applicability (**Figure 7**). Cross validation of clinical radiomic models using repeated k-fold CV, average accuracy and standard deviation of clinical–radiomics model are 90.23% and 8.45%, respectively, which demonstrate the stability of the model. The Hosmer–Lemeshow tests were performed to assess the goodness-of-fit of the clinical-radiomics model (χ^2^ = 2.287, P=0.971), and the results indicated that the model had good calibration.

## 4 Discussion

ERM has gradually been phased out because of the long operative time and attendant postoperative complications (13). Owing to the recent and ongoing advancements in minimally invasive techniques, IM-SLNB or video-assisted IMN dissection can provide less invasive means of IMN metastasis status evaluation. However, the learning curve for minimally invasive surgery is beset by challenges, including operative time, SLNB false-negative rate, and sensitivity (21). Several studies have confirmed that the Memorial Sloan Kettering Cancer Center nomogram based on nine clinicopathological variables, such as age, tumor size, tumor type, tumor location, lymphovascular invasion, multifocality, histological grade, ER status, and PR status, can be utilized to accurately and noninvasively assess the risk of ALN-SLNB metastasis in patients with breast cancer (15, 18).

The incidence rate of IM-SLN metastasis was 17.0% to 33% for patients clinically diagnosed with ALN positivity, and 10% for ALN-negative patients based on IM-SLNB (19, 20). And tumor size, tumor location, lymphovascular invasion, and number of positive ALNs were independent factors influencing IM-SLN metastasis (19, 20). Therefore, this clinical prediction model can also predict the IMN metastasis status before surgery (19–22). Huang et al. (22) also found that the medial quadrant location, PR positivity, and ALN metastasis were predictive factors for IMN metastasis. Our model focused on predicting the risk of IMN metastasis in Chinese patients with breast cancer, and our results support its accuracy, with a statistically significant AUC of 0.913.

Radiomics is a high-throughput data-mining technique that extracts quantitative image features from various imaging modalities such as ultrasonography, computed tomography, MRI, and positron-emission tomography, etc., enabling the conversion of image information into extractable data. Subsequently, these radiomics data can be further analyzed and applied to clinical decision-making systems. The clinical implementation of radiomics models offers a novel approach to establishing lymph node metastasis prediction models to enhance the accuracy of diagnosis, prognostication, and prediction (23–25). Currently, numerous radiomic models based on mammography, ultrasound, and breast MRI modalities exist for breast cancer ALN metastasis prediction, with AUC values ranging from 0.799-0.920. These findings underscore the potential of radiomics models for preoperative ALN metastasis prediction (26–29).

We retrospectively analyzed the clinicopathological characteristics and preoperative DCE-MRI radiomics features of patients with breast cancer to establish distinct clinical and radiomics models to perform noninvasive preoperative prediction of IMN metastasis status more effectively, and combined the two models into a comprehensive prediction tool. The aim was to explore an optimal prediction model for IMN metastasis that could guide risk assessment and individualized treatment strategies. Among the various MRI sequences available, DCE is widely regarded as the optimal sequence for identifying primary breast tumors (15). Therefore, we extracted radiomics information from DCE-MRI scans. Considering the varying evaluation effects of different DCE sequences, study has proved that the second-phase (CE2) image performed best when acquired 60-90 s after contrast-medium administration, because it provided the strongest contrast between the tumors and surrounding tissues (30). Similarly, our radiomics model was developed based on the ROI delineated from the second phase of preoperative DCE-MRI to further extract and screen the relevant radiomic features. The AUC value for this radiomics model utilizing preoperative DCE-MRI information alone was 0.831, which indicated accurate prediction of IMN status among patients with breast cancer.

As mentioned before, the findings of our study indicate that both the clinical model based on clinicopathological features and the radiomics model based on DCE-MRI features had good predictive ability for the risk of IMN metastasis. We compared the ROC performance of the clinical and radiomics models to determine the optimal prediction model for predicting the IMN metastasis status. Although the AUC of the clinical model was higher than that of the radiomics model (0.913 vs. 0.831), the Delong test revealed no statistically significant difference (p=0.134). To further ascertain the more effective model for IMN metastasis risk prediction, a combined model was developed by integrating the clinicopathological and radiomic features, and its effectiveness was validated. A nomogram that incorporates clinical factors and radiomics features provides clinicians with visual support for decision-making and highlights the predictive potential of the fusion model (31). The fusion model also demonstrates promising prospects for predicting lymph node metastasis in breast cancer. Preoperative prediction of axillary lymph nodes status has been achieved using radiomics and combined models (25, 29, 30, 32).

Although preoperative prediction of ALN status has been achieved using radiomics and combined models, there is a dearth of research on IMN metastasis status prediction. In this study, we combined clinicopathological features, viz. tumor location, PR status, and N stage, with radiomics features to establish a clinical–radiomics model. This model achieved a higher AUC value of 0.964 compared to the clinical and radiomics models alone. Internal validation of the combined model further confirmed its good discriminatory ability (Hosmer–Lemeshow goodness-of-fit test: χ²=2.287, P=0.971). Therefore, our clinical–radiomics model was deemed the optimal predictive tool for risk assessment of IMN metastasis. These results show that incorporating radiomics into predictive models can improve the accuracy of IMN metastasis risk assessment and aid in identifying patients who may benefit from avoiding overtreatment or receiving additional interventions such as IMN radiotherapy (IMN-RT). To facilitate clinicians’ predictions of IMN metastasis risk based on clinicopathological factors and preoperative MRI features, we developed a nomogram that visualizes the results and confirms the superior predictive value of our combined model; however, it cannot replace the individualized decision-making for IMN-RT. The potential utility of this prediction model may lie in identifying patients at very low or high risk of IMN metastasis who would derive the maximum benefit from its implementation.

Our study has certain limitations. First, this single-center retrospective study included a relatively small sample size and a relatively low number of patients with IMN metastasis. Therefore, we employed data integration techniques to construct a predictive model that was internally validated by 1000 rounds of bootstrapping. Second, the study lacked external validation, which is a crucial step in establishing the credibility of the predictive model; multicenter validation is imperative to enhance the level of evidence for clinical application. Additionally, the utilization of only one imaging modality resulted in a limited number of extracted radiomic features. If more imaging modalities can be combined, the feature library can be further expanded to obtain more valuable radiomics information.

## Data Availability

The raw data supporting the conclusions of this article will be made available by the authors, without undue reservation.
